# Channel-Agnostic Training of Transmitter and Receiver for Wireless Communications

**DOI:** 10.3390/s23249848

**Published:** 2023-12-15

**Authors:** Christopher P. Davey, Ismail Shakeel, Ravinesh C. Deo, Sancho Salcedo-Sanz

**Affiliations:** 1 School of Mathematics, Physics and Computing, University of Southern Queensland, Springfield, QLD 4300, Australia; chris.davey@unisq.edu.au (C.P.D.); sancho.salcedo@uah.es (S.S.-S.); 2 Spectrum Warfare Branch, Information Sciences Division, Defence Science and Technology Group (DSTG), Edinburgh, SA 5111, Australia; ismail.shakeel@defence.gov.au; 3 Department of Signal Processing and Communications, Universidad de Alcalá, Alcalá de Henares, 28805 Madrid, Spain

**Keywords:** deep learning, channel free training, wireless communications, over-the-air training, neural networks

## Abstract

Wireless communications systems are traditionally designed by independently optimising signal processing functions based on a mathematical model. Deep learning-enabled communications have demonstrated end-to-end design by jointly optimising all components with respect to the communications environment. In the end-to-end approach, an assumed channel model is necessary to support training of the transmitter and receiver. This limitation has motivated recent work on over-the-air training to explore disjoint training for the transmitter and receiver without an assumed channel. These methods approximate the channel through a generative adversarial model or perform gradient approximation through reinforcement learning or similar methods. However, the generative adversarial model adds complexity by requiring an additional discriminator during training, while reinforcement learning methods require multiple forward passes to approximate the gradient and are sensitive to high variance in the error signal. A third, collaborative agent-based approach relies on an echo protocol to conduct training without channel assumptions. However, the coordination between agents increases the complexity and channel usage during training. In this article, we propose a simpler approach for disjoint training in which a local receiver model approximates the remote receiver model and is used to train the local transmitter. This simplified approach performs well under several different channel conditions, has equivalent performance to end-to-end training, and is well suited to adaptation to changing channel environments.

## 1. Introduction

**Figure 1:**
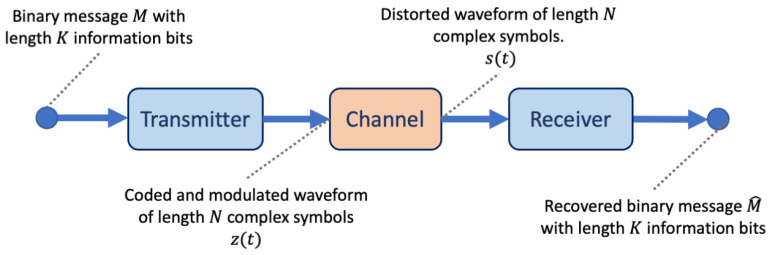
A simplified wireless communications system comprising a transmitter, a channel environment, and a receiver. The transmitter takes the input message block, then performs encoding and modulation prior to sending it over the channel. The channel distorts the waveform; such distortions can include noise and fading. The receiver must detect and filter the content of the received waveform, then demodulate and decode the data in order to recover the original message.

The primary goal of a wireless communications system is to transmit a message over-the-air (the channel environment) to a receiver such that the message can be recovered without error. However, the channel environment causes distortions in the transmitted signal that impede perfect recovery of the message. To improve message recovery, communications systems are designed with multiple signal processing blocks and with complementary components between the transmitter and receiver for each stage (coding/decoding, modulation/demodulation, filtering/detection). Figure 1 illustrates a simple wireless communications system comprising a transmitter, channel, and receiver. Each of these stages is traditionally designed and optimised independently while assuming a fixed mathematical model of the channel.

More recently, deep learning (DL) in wireless communications systems has been applied to jointly optimise functions for the transmitter and receiver over an assumed channel model [1]. Such approaches offer an alternative to the block design of communications systems, and may achieve better performance in complex channels without a formal model [1]. The supervised learning procedure enables the transmitter to learn complex domain symbols, thereby maximising the ability of the receiver to de-noise and map soft channel outputs to the original message. The DL auto-encoder (AE) architecture is a proven approach for application to automatic feature learning, and is coupled with noise distortions during learning to enable the decoder component of the architecture to learn robust features for de-noising and estimation [2].

During training, the perturbations provided by an assumed channel model help the transmitter (encoder) to learn robust features through the process of backpropagation. Backpropagation communicates the loss at the receiver (decoder) by applying the chain rule with respect to the training loss function, which requires a differentiable channel function to pass the gradients from the receiver to the transmitter. The true channel environment prevents backpropagation between the transmitter and receiver, representing a key challenge in the over-the-air training of AE for wireless communications systems.

Research into over-the-air learning for wireless communications systems has demonstrated approaches in which the transmitter and receiver can be trained in a disjoint manner. DL approaches which leverage the AE architecture to model the transmitter, channel, and receiver have approached the problem by training an end-to-end system offline with an assumed channel model (Joint Learning) and tuning the receiver model online against the pretrained transmitter [3] (Receiver Tuning). During the tuning phase, the transmitter is not updated under the true channel conditions, preventing improvement of the code learned by the transmitter during the tuning phase. Thus, any improvement under the new channel depends on the adaptation of the receiver.

The transmitter learns a code that relies on the properties of the channel environment, which are modelled during training. The Joint Learning process results in a code that maximises the mutual information between the transmitted (channel input) and received (channel output) symbols through direct observation of the channel [4]. In contrast, conventional coding methods counteract channel effects such as fading by introducing redundant symbols (diversity) or using estimates of fading coefficients (channel state information) for precoding at the transmitter or correction at the receiver [5]. DL techniques have demonstrated the ability to learn accurate estimates for channel state information, and have been applied to correction and signal detection at the receiver [6,7]. The application of DL to channel modelling has led to the adoption of generative adversarial network (GAN), which can learn to emulate the stochastic channel environment [8], motivating the potential application of DL to either explicitly model the channel environment or implicitly extract channel state information during over-the-air learning (OAL).

Two methods of extending DL to OAL involve feedback from the receiver to enable learning a proxy of the channel, thereby permitting backpropagation between the transmitter and channel model [9,10,11,12] (Channel Approximation). Another approach is Gradient approximation, in which the gradient at the transmitter is approximated through variants of finite difference approximation or reinforcement learning (policy-gradient learning) [13,14,15,16]. Additional methods involve multi-agent approaches such as Collaborative Agent Learning coordinated by specific training protocols [17], which is able to include a variety of learning algorithms other than DL. Both the channel and gradient approximation approaches have demonstrated equivalent performance to the end-to-end joint learning approach [11,13,14], while the Collaborative Agent Learning method has demonstrated performance close to conventional codes [17].

In this paper, we refer to Receiver Tuning, Channel Approximation and Gradient Approximation as methods of Disjoint Learning and regard these as separate from the Collaborative Agent Learning approach. We present an additional method of Disjoint Learning, “Learning through Imitation”, that is situated between Channel Approximation and Gradient Approximation, where a local channel/receiver model is developed using estimates from the actual receiver to imitate the behaviour of the channel/receiver at the transmitter side. This enables the application of supervised learning to train the transmitter using backpropagation. This approach does not model the channel directly; instead, it learns to mimic the errors made by the remote receiver and acts as a proxy for the remote receiver model. We use simulation to produce equivalent results to the end-to-end Joint Learning approach first demonstrated in [1] and show that this method outperforms receiver tuning. To show that the local receiver model approximates the remote receiver model, we compare the process of learning without feedback to that of learning with feedback, and demonstrate that learning through imitation exceeds the performance of learning without feedback.

Therefore, with the aim of providing a novel method for channel-agnostic over-the-air training of both transmitter and receiver for resilient wireless communications, the primary objectives of this study are as follows:

- To propose a novel over-the-air training method and develop machine learning enabled coding and modulation schemes for the transmitter and the receiver without an assumed channel model.
- To develop a Disjoint Learning method that uses a transmitter-side (local) channel/receiver to imitate the learning process of the remote receiver and enable supervised learning of the transmitter through backpropagation.
- To demonstrate that the performance of the proposed Disjoint Learning method is equivalent or better than the fully connected architecture.
- To show that the proposed method achieves significant performance improvements against the Receiver Tuning training method.

The rest of this paper is organised in the following way: Section 2 provides a brief overview of related work; Section 3 describes our proposed model, training, and simulation methods; Section 4 presents results for the proposed method and provides a discussion of the results and modelling approach; and Section 5 draws conclusion and proposes future directions for investigation.

## 2. Background and Related Works

The canonical application of DL for the joint learning of a wireless communication system is presented in [1]. An AE transmitter and receiver model was shown to perform equivalently to short uncoded and Hamming(7,4) coded messages (K=4 information bits and N=7 code bits) on the Additive White Gaussian Noise (AWGN) channel [1]. The authors observed the relationship between the choice of energy constraint and constellation learned by the transmitter. The influence of the channel on the system was shown by training two pairs of transmitter and receiver AEs on an interference channel. The transmitters learned to counteract the interference channel by developing orthogonal codes [1]. It is acknowledged that both symbol-wise AE (classification mapping code word to message) and bit-wise AE (modelled as *K*-bit outputs) are limited in their application to smaller codes due to the dimensionality of a possible $2^{K}$ messages for *K* information bits. The joint learning approach demonstrates the inclusion of an assumed channel transfer function in the design of the network, and must be trained offline. This prevents joint optimisation on the true channel environment.

Receiver tuning, inspired by transfer learning, was carried out after the joint learning phase and used to update the trained receiver on the true channel in [3]. The resulting system was compared with differential quadratic phase shift keying (DPSK) in both simulated AWGN and over-the-air channels. The simulated channel included impairments for timing, phase, and frequency offsets, while the receiver model was developed to correct for these distortions before decoding [3]. Receiver tuning was demonstrated to improve the performance of the AE over the end-to-end model, but did not improve upon the DPSK modulation. However, the approach demonstrated a practical way forward in tuning AE models over-the-air. The primary disadvantage of receiver tuning is that the transmitter remains fixed during the tuning phase and does not adapt to the true channel distortions compensated by adaptation at the receiver.

Methods for disjoint learning emerged to address the limitations of receiver tuning and permit over-the-air training of both transmitter and receiver models. Channel approximation methods using GANs [18,19] have been applied to train a proxy for the channel in response to feedback and enable the transmitter to be trained with backpropagation through the generator channel model [9,10,11,12]. In [9], a channel model inspired by the GAN approach was trained to approximate the channel response directly, and the transmitter was updated by alternating backpropagation phases between channel and receiver loss. A local receiver (acting as the discriminator) is required in order to enable end-to-end learning for the transmitter, and leverages the channel model for backpropagation. This approach was extended in [10] to leverage a separate discriminator network, while a variational neural network was incorporated in [20] to describe the channel distribution in the generator. The variational method has been shown to better approximate the variance of the channel response for a range of channels in comparison to the previous method based on mean squared error (MSE) loss [10]. These approaches introduce a separate training procedure to train the generator in order to approximate the true channel environment.

In [21], a conditional GAN was trained to approximate the AWGN and Rayleigh fading channels conditioned on the pilot symbols in [11], then used to optimise a transmitter and receiver for symbol classification. The channel model was shown to approximate the AWGN perturbations for a quadrature amplitude modulation (QAM) of sixteen symbols [11]. The performance of the system was shown to be equivalent to a Hamming(7,4) code over AWGN and to perform similarly to coherent detection in a Rayleigh fading channel [11]. The approach was later combined with convolutional neural network (CNN) modules for bit-wise estimation for longer message lengths in [12]. A simple feed-forward GAN was compared with 4-QAM Hamming(7,4) code under AWGN. A CNN-GAN was compared to a convolutional code in the Rayleigh fading and selective-frequency channels in [12]. Performance in each channel was shown to be close to the conventional methods, and the importance of the pilot symbols was empirically demonstrated in the selective frequency channel [12]. The GAN approach introduces complexity to the training procedure due to the need to alternate between training the discriminator and generator as well as between the transmitter and receiver training phases.

A one-shot training approach for a conditional GAN was adopted in [22] to simplify the training procedure. It was used to train an AE model that supports longer messages lengths by combining the AE with bit-interleaved coded modulation (BICM) and an outer low-density parity-check (LDPC) code [22]. Comparison against a 16-QAM baseline and a AE-GAN trained on a simulated AWGN channel were made, as well as with a AE-GAN trained over-the-air and the reinforcement learning (RL)-based approach described in [16]. The AE-GAN trained on the true over-the-air channel environment demonstrated improved performance over the same approach trained on a simulated channel [22]. The approach required two stages, with the GAN first trained independently of the AE and later applied to train the AE on the receiver side. While it was suggested that the GAN framework could be used to model the channel without prior knowledge, the authors reported difficulties in training the GAN considering the presence of carrier frequency offset (CFO), which prevented the GAN from converging [22].

A separate channel model is not a necessity for optimisation of the transmitter and receiver models. Other approaches have focused on gradient approximation methods to support backpropagation at the transmitter. A finite difference gradient approximation method, Simultaneous Perturbation Stochastic Approximation (SPSA), was applied in [13]. The transmitter symbols were perturbed multiple times with a given noise distribution and the receiver errors were collected for each point and applied to approximate the gradient at the transmitter [13]. The model was demonstrated to be equivalent to uncoded quadrature phase-shift keying (QPSK) in AWGN and very close to theoretical uncoded QPSK in Rayleigh block fading channels. In addition, it was shown to be comparable in performance to the end-to-end AE described in [1]. The above process is computationally expensive. Results are taken from an average of 250 independent models; each time the gradient is approximated, the transmitter outputs are combined with a small perturbation vector and the receiver loss is calculated for each [13]. Due to the amount of sampling required to approximate the gradient, this method would encounter difficulty scaling to more complex transmitter models or longer message sequences.

An alternate gradient approximation approach proposed in [14] is based on policy gradient (PG) approximation. Such methods are applied in deep RL; an agent learns to exploit actions in response to the environment, resulting in the highest expected reward [23,24]. In [14], a penalty signal is provided by the receiver loss. The transmitter is trained to minimise the loss without an explicit model of the channel environment. Learning is achieved by alternating between the training of the receiver and the transmitter. This approach does not require a local proxy for the receiver, as the gradient can be estimated directly from the loss signal calculated for perturbations of the complex symbols learned at the transmitter. This process generates a stochastic sampling scheme equivalent to RL “policy” exploration [14]. The approach was evaluated in both AWGN and Rayleigh fading channels. In the latter, the receiver network was modified with a prior assumption of the channel distortion to learn estimates of the fading coefficients and reverse the fading prior to the discriminative layers of the network [14]. While the authors indicated that the training procedure requires more iterations than the end-to-end method, their evaluation demonstrated equivalent performance to end-to-end AE in both channels [14]. The method was tested over-the-air with software defined radio (SDR) in [15,16] and had a lower error rate in comparison to conventional codes. Both of these sources indicate that the method requires an extended training duration and that the variance of the receiver loss negatively impacts the convergence of the gradient at the transmitter [15,16]. To address the long training time, it has been proposed to pretrain the network offline and perform online tuning of both the transmitter and receiver [15].

The deep deterministic policy gradient (DDPG) approach was applied in [25] to address the issues around convergence described in [14] by applying both a “replay” buffer (sometimes termed an “experience” buffer) and a soft update rule used to transfer learned weights between a duplicate transmitter and an accompanying critic network. This method was reported to outperform the alternating algorithm in both Rayleigh and Rician fading channels [25]. The addition of the replay buffer requires additional memory to store previous receiver losses, and the additional critic network increases the complexity of the training algorithm in a trade-off with the improved learning at the transmitter.

The problem of training both the transmitter and receiver has been framed as a collaborative agent problem. These types of approaches are interesting because they can coordinate training between different types of learning algorithms for the transmitter and receiver. A hybrid approach called Collaborative Multi-Agent Learning was presented in [26]. This method trains a neural network transmitter using RL to learn the symbol constellation and a k-means clustering receiver to determine the number of symbols and estimate the message. A transmitter (Tx A) outputs a modulation for a given preamble, then transmits to a receiver (Rx B) over an AWGN channel, which produces an estimate of the message; this estimate is relayed through the second transmitter (Tx B) to a receiver on the originating side (Rx A), which is used to estimate a loss signal for the original transmitter (Tx A) [26]. This echo procedure has been shown to produce varying-order modulations under different training regimes for noise and energy constraints [26]. However, it did not achieve comparable results to the baseline QAM modulation [26]. The echo procedure is complex in that it requires two pairs of transmitter and receivers; each pair iteratively swaps between sending the original message to update each transmitter.

An echo protocol with a private preamble was applied in [17]. Pairs of collaborating agents share information about the learning task, and the difficulty of learning increases as less information is exchanged [17]. The authors asserted that their proposed echo protocol with private preamble enables learning of different types of agents and minimises the amount of information sharing between agents [17]. The method was demonstrated to perform similarly to QPSK under AWGN as well as in over-the-air experiments [17]. Both sources [17,26] leveraged a similar approach in defining transmitter and receiver pairs as agents during training, and both applied RL to train the transmitter. While neither approach outperforms conventional codes, the technique of using the receiver estimate as an echo is of interest for our method. Our proposed method learns to imitate the feedback from the remote receiver estimate, which includes the errors made during training.

Regularisation in DL seeks to reduce the bias of the network towards training data. It achieves this through reducing the complexity of the model during training [27]. Mechanisms include penalising weights (weight normalisation and averaging), perturbation of inputs (such as the transformations applied to images in computer vision), learning normalisation of activations (batch and layer normalisation), perturbation of network structure (such as drop-out), and training algorithms (such as stochastic gradient descent (SGD)). The use of incorrect labelling has been shown to provide regularisation for classification tasks [28]. This method makes use of a small noise rate to modify the ground-truth label of each class by selecting from weighted alternatives [28]. It had been shown to slow convergence and reduce overfitting of the model during training [28]. The authors used a fixed noise rate parameter and showed improvements when training reference models on several computer vision benchmarks [28]. While the noise rate is not decreased during training, this approach is relevant to our proposed method. Early in the learning process, the remote receiver yields a less accurate estimate which corresponds to a higher loss. The estimates become more accurate during the learning process, and the loss gradually decreases as learning progresses. The local channel/receiver is trained to imitate the estimates output by the remote receiver. In this manner, the learning process is comparable to training against noisy classification targets where the noise rate decreases over time. The purpose is to enable the local channel/receiver to learn from the noisy estimation process at the remote receiver.

The surveyed approaches for learning wireless communications systems have included joint learning, disjoint learning, and collaborative agent learning. Our focus is on joint and disjoint learning, with the the focus of this literature review on methods for training AE neural network models. Our proposed method differs from the GAN and RL methods surveyed above. In comparison to GAN methods, our method does not learn an explicit channel generator model and does not require a discriminator model during training. Instead, a local channel/receiver model is trained to imitate the remote receiver model. In comparison to RL-based methods, we do not perform gradient approximation; hence, we do not require multiple perturbations during the forward pass to estimate the gradient at the transmitter, and do not require additional support from methods such as a “replay” buffer to address variation in the loss estimate. Instead, the local channel/receiver model acts as a proxy for the remote receiver model to support end-to-end backpropagation at the transmitter. While we do leverage the remote receiver estimate as feedback, which is somewhat similar to the echo protocol in collaborative agent learning, we do not require additional coordination protocols for multiple agents and do not train transmitter/receiver pairs. Instead, our method trains a local proxy on the transmitter side against the feedback of estimates from the remote receiver. Our simplified approach removes the need for channel generative modelling, gradient approximation, or coordination protocols.

## 3. Methodology

In this section, we describe our proposed approach and the channel environment simulation used for training and evaluation of the resulting transmitter and receiver models. Section 3.1 briefly describes the communications system, Section 3.2 outlines our proposed method and training approach, and Section 3.3 describes the channel environments used for simulation during training and evaluation.

### 3.1. System Description

A wireless communications system aims to communicate a *K* bit message *x*. The transmitter converts the message into an optional code word of length *N*, and converts the message (or code) into a set of modulated symbols. It then combines the modulation with a carrier wave to transmit a set of complex values z(t)∈C with t=1…T timesteps and applies a filter to prevent inter-symbol interference. These values are transmitted through a channel environment that causes distortions including noise and fading effects. The channel environment is represented in our simulations as a channel transfer function r(t)=h(z(t)). The received signal r(t) is filtered and imperfections are corrected, then it is demodulated and decoded to produce an estimate for the original *K* bit message *y*. In a wireless communications environment, there are mismatches at the transmitter and receiver in the timing, phase, and frequency between the transmitted signal z(t) and received signal r(t). Such imperfections can be simulated with the channel transfer function. However, for this work we assume perfect synchronisation and do not perform corrections for these offsets at the receiver, nor do we perform filtering at the transmitter and receiver. Our focus is on training a local transmitter DL model to perform modulation and coding, simulating the physical channel external to the DL models, and training the remote receiver DL model to estimate the original message.

### 3.2. Proposed Approach

**Figure 2:**
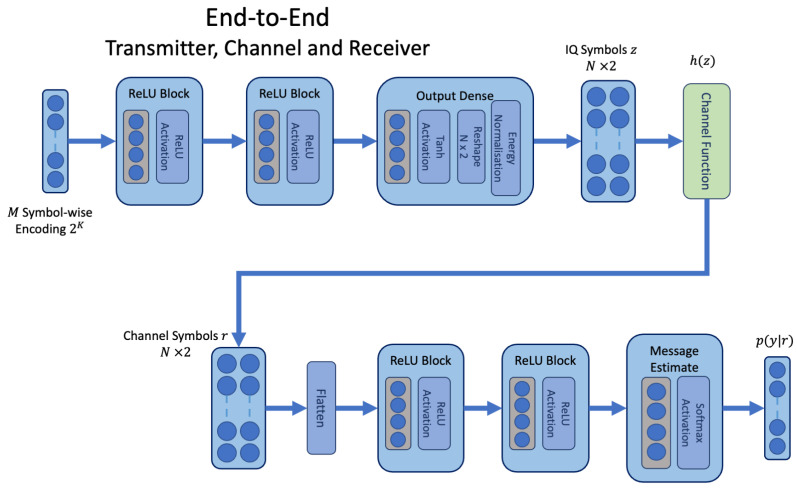
The end-to-end network architecture, where an assumed channel transfer function is defined as a layer within the network architecture.

We start with the joint learning of an end-to-end AE model, similar to the architecture described in [1]. This model, shown in Figure 2, consists of a transmitter neural network $Tx(x,\theta_{t})$ with weights $\theta_{t}$ and a receiver neural network $Rx(r,\theta_{r})$ with weights $\theta_{r}$ linked by an assumed channel function h(z). The main paths of both networks consist of feed-forward dense modules followed by a rectified linear unit (ReLU) activation [29]. In the transmitter, a *tanh* activation is applied prior to an energy normalisation layer. The modelling approach focuses on small block codes using a symbol-wise representation; hence, input messages of *K* bits are one-hot encoded as $2^{K}$ words prior to presentation to the transmitter. A one-hot encoded vector *x* has length $2^{K}$ and contains a one at the index corresponding to the selected message and zeroes in all other index positions.

A set of complex soft values z∈C are output by the transmitter neural network prior to the channel, and represent the code of length *N*; these are defined as two real numbered vectors for the in-phase and quadrature IQ coordinates within the network $z=Tx(x,\theta_{t})$. Inputs to the receiver result from application of a channel transfer function r=h(z), and the receiver converts these channel symbols back to a probability distribution over each message $p\left(y|r\right)=Rx\left(r,\theta_{r}\right)$. The probability vector contains an estimate for each of the possible $2^{K}$ messages using the *softmax* activation. This activation function takes as input the vector produced by the final dense layer *f* of the receiver neural network, which has $2^{K}$ linear units. The *softmax* activation is defined as $p\left(y_{i}|r\right)=\exp\left(f_{i}\right)/\sum_{j}^{2^{K}}\exp\left(f_{j}\right)$; the summation in the denominator ensures that the outputs sum to 1, and corresponds to a probability density. The model performs classification by taking the index with the highest probability as the index for the corresponding *K* bit word in the lookup table containing all possible words $\hat{M}=\arg\max p\left(y_{i}|r\right)$.

In the canonical AE, the transmitter model, assumed channel function, and receiver model are connected such that training can be carried out end-to-end with backpropagation. Backpropagation consists of a forward pass $p\left(y_{i}|r\right)=Rx\left(h\left(Tx\left(x,\theta_{t}\right)\right),\theta_{r}\right)$ and a backward pass that updates the weights at each layer by calculating the derivative with respect to the loss by application of the chain rule. The model is trained to minimise the cross-entropy loss between the true and estimated message labels in Equation (1). The expression $p(y_{true})$ indicates the target one-hot encoded vector for the true message presented during training. Typically, the network is presented with batches of data and the loss is averaged over the entire batch.

(1) $$L\left(p\left(y_{true}\right),p\left(y|r\right)\right)=-\sum_{i=1}^{2^{K}}p\left(y_{i}\right)\log p\left(y_{i}|r\right)$$

The backward pass calculates the gradients for the weights in the network. For the receiver, the backward pass applies the chain rule between the receiver network model and the loss function in Equation (2), and updates the weights by taking a small step in the direction of the gradient $\theta_{r}=\theta_{r}-\eta \nabla_{\theta_{r}}$, where η represents a small learning rate constant. For the transmitter, the backward pass includes the gradients from the receiver as well as the gradient for the channel function with respect to the transmitter model, and updates the transmitter weights accordingly: $\theta_{t}=\theta_{t}-\eta \nabla_{\theta_{t}}$. In stochastic gradient descent, the gradient is calculated over the batch and several enhancements to the method add features, for instance, momentum to dynamically control the step size during learning, or adaptive learning rates for different parameters of the model, such as in the Adam optimizer [30].

(2) $$\nabla_{\theta_{r}}=\frac{\partial Rx(r,\theta_{r})}{\partial \theta_{r}}\frac{\partial L\left[p\left(y_{true}\right),Rx\left(r,\theta_{r}\right)\right]}{\partial Rx(r,\theta_{r})}$$

(3) $$\nabla_{\theta_{t}}=\frac{\partial Tx(x,\theta_{t})}{\partial \theta_{t}}\frac{\partial h(Tx\left(x,\theta_{t}\right))}{\partial Tx(x,\theta_{t})}\nabla_{\theta_{r}}$$

During receiver tuning, the transmitter and receiver models are detached from the channel layer and the receiver model is updated via backpropagation while the transmitter remains frozen. Therefore, receiver tuning does not require differentiation through the channel function.

The architecture we apply in disjoint learning consists of a disconnected transmitter model $Tx(x,\theta_{t})$ and a receiver model $Rx(r,\theta_{r})$, and we simulate a channel transfer function h(z) separately from both models so that the channel does not participate in backpropagation. This is to simulate the process of over-the-air learning. In over-the-air learning, the channel may take on more complex behaviour than is captured by the assumed mathematical channel function. Therefore, training from the true channel is desirable, as it can permit the network to learn a coded modulation that is optimised for the true channel environment. As described in Section 2, the current approaches to disjoint learning achieve backpropagation at the transmitter by either explicitly learning the channel or by approximating the channel gradient. Instead of learning the channel directly, we rely on a local proxy for the remote receiver at the transmitter side, which we use to perform backpropagation without training an explicit channel model.

**Figure 3:**
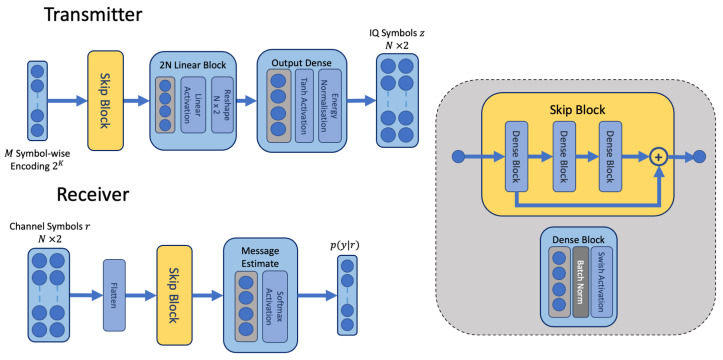
The architectural blocks for disjoint learning of transmitter and receiver. The same architecture is applied in both local and remote receivers. A channel transfer function is not assumed as part of the model.

Before describing the training method, we first describe the structure of the network architectures for the transmitter and receiver models. The transmitter and receiver neural network architectures contain a series of fully connected dense blocks, similar to the end-to-end AE; however we add skip connections in the main path of each network. This architecture is illustrated in Figure 3. The skip connections, described as a “Skip Block” in the figure, assist backpropagation and combine features learned in the earlier hidden layers with the upper hidden layers [31]. In addition to the effect on backpropagation, skip connections are indicated to learn an ensemble of networks [32]. Each skip block is comprised of several dense blocks containing batch normalisation [33] and a nonlinear swish activation [34]. Input to and output from the transmitter follows the same principle as the end-to-end architecture, as does the input and output from the receiver. The layers, unit sizes, and groups within the transmitter are described in Table 1, while the receiver is described in Table 2. The dimension of the networks was arrived at through a manual process; while it is possible to use automated procedures for finding the best dimensions, such processes often tend to be computationally demanding and require a long duration. We chose a manual stepwise approach for simplicity, gradually increasing the dimensions of each layer by powers of 2. It is interesting to note that learning shorter codes appears to be more challenging than learning codes with longer lengths, requiring a larger dimension of the intermediate dense layer within the skip block for the 4/7 code rate as opposed to the uncoded 8 bit message.

**Figure 4:**
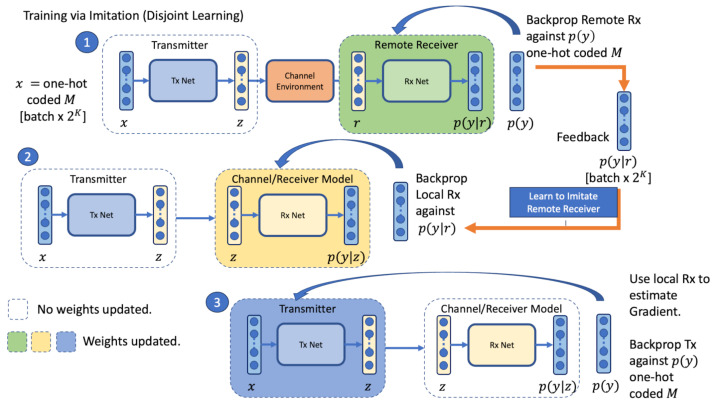
The three stages of the training procedure consist of a forward pass through the transmitter $z=Tx(x,\theta_{t})$, channel r=h(z), and remote receiver $p\left(y|r\right)=Rx\left(r,\theta_{r}\right)$. The remote receiver estimates p(y|r) are obtained through the feedback channel. The second stage trains the local channel/receiver model using the KL divergence loss between the local channel/receiver estimates p(y|z) and remote receiver estimates p(y|r). The third stage trains the transmitter using the local receiver as a proxy to enable end-to-end backpropagation.

The training procedure is illustrated in Figure 4, which shows the three stages of the proposed disjoint training regime. This approach consists of training three models: a local transmitter model $Tx(x,\theta_{t})$, a local channel/receiver model $Rx_{L}\left(z,\theta_{l}\right)$, and a remote receiver model $Rx_{R}\left(r,\theta_{r}\right)$, separated by a channel h(z) which is not connected to the network models. The local channel/receiver model does not receive inputs *r* from the simulated channel; instead, it takes its inputs directly from the output of the transmitter model $z=Tx(x,\theta_{t})$. During training a feedback channel is required, allowing the average value to be captured for the remote loss per batch along with remote estimates p(y|r) for each item in the batch. Only one network is trained at each stage.

To generate the same sequences of random messages in each training iteration, both sides are initialised with the same random seed at the start of each batch. In the first stage, the local transmitter remains frozen and provides the forward pass for the batch of symbols $z=Tx(x,\theta_{t})$ that are sent to the remote receiver over the simulated channel r=h(z). The remote receiver is trained with SGD against the cross-entropy loss between the true and estimated message labels in Equation (1). The remote receiver probability estimates p(y|r), along with the mean loss, are sent over the feedback channel to the local transmitter. In the second stage, the local receiver is trained to imitate the remote receiver using the Kullbach-Leibler (KL) divergence loss in Equation (4). The aim is to minimise the difference between the estimated probabilities at the remote receiver $p\left(y|r\right)=Rx_{R}\left(r,\theta_{r}\right)$ given the channel symbols, and the estimated probabilities at the local channel/receiver $p\left(y|z\right)=Rx_{L}\left(z,\theta_{l}\right)$ given the local transmitter symbols *z*. This allows the local channel/receiver to learn how to act as a proxy for the remote receiver without an explicit model of the channel by imitating the estimation produced at the remote receiver. As indicated by Equation (5), the gradient for the connected local transmitter Tx and the local channel/receiver $Rx_{L}$ can be described as a weight update for the combined weights $\theta_{t,l}$ of the end-to-end connected model $\theta_{t,l}$. Because the feedback channel provides p(y|r) from the remote receiver, the backpropagation at the transmitter side is no longer dependent on an assumed channel function.

(4) $$L_{stage2}\left[p(y|r),p(y|z)\right]=\sum_{i=1}^{2^{K}}p\left(y_{i}|r\right)\log\frac{p(y_{i}|r)}{p(y_{i}|z)}$$

(5) $$\nabla_{\theta_{t,l}}=\frac{\partial Tx(x,\theta_{t,l})}{\partial \theta_{t,l}}\frac{\partial Rx_{L}\left(Tx\left(x,\theta_{t,l}\right),\theta_{t,l}\right)}{\partial Tx(x,\theta_{t,l})}\frac{\partial L_{\mathrm{stage}2}\left[p\left(y|r\right),Rx_{L}\left(Tx\left(x,\theta_{t,l}\right),\theta_{t,l}\right)\right]}{\partial Rx_{L}\left(Tx\left(x,\theta_{t,l}\right),\theta_{t,l}\right)}$$

Both Stage 1 and Stage 2 make use of a larger batch size than the third stage, which we set at 320 samples in stages 1 and 2 and 32 samples in Stage 3. In the third stage, a forward pass through the transmitter is made for a new batch. The local channel/receiver is used to calculate the cross-entropy loss against the true messages $L\left[p(y),p(y|z)\right]$. The local channel/receiver estimates are conditioned on the output of the local transmitter *z*, rather the output of the simulated channel *r* as is the case on the remote receiver. Updates resulting from the backpropagation process occur only on the local transmitter, as the local channel/receiver weights are frozen during this step. The label noise introduced in the second stage enables the transmitter to learn appropriate IQ symbols to assist the remote receiver in the labelling task. To demonstrate that this approach has an effect, we performed training with no feedback, in which the second stage of the algorithm updates a local receiver model against the true message using the cross-entropy loss instead of optimising toward the remote distribution. In Section 4, we demonstrate that training the local channel/receiver to imitate the remote receiver produces an observable difference in performance in comparison to the same algorithm without feedback.

Energy normalisation is applied to constrain the output of the transmitter such that ${\left||x|\right|}_{2}^{2}\leq 1$, as defined in Equation (6), where the learned code x(t) with length *L* is divided by its scaled Euclidean norm to produce the transmit symbols z(t).

(6) $$z\left(t\right)=\frac{x(t)}{\sqrt{\sum_{i=1}^{L}x{\left(i\right)}^{2}/L}}$$

Each of the networks are trained using the Adam algorithm [30], and we combine stochastic weight averaging (SWA) [35] with a cyclical learning rate schedule [36] which oscillates between learning rates of 0.0001 and 0.001. In this work, we simulate the channel transfer function as described in Section 3.3. This allows the signal to noise ratio (SNR) dB to be randomised during training of the remote receiver; however, in an over-the-air setting, the SNR dB parameter cannot be set explicitly.

### 3.3. Simulated Channel Functions

Comparisons between models were made in simulated channel environments for AWGN, Rayleigh, and Rician fading as well as for an AWGN channel with nonlinear amplifier effects, namely, power amplifier Additive White Gaussian Noise (PA-AWGN). In the AWGN channel, the transfer function adds a noise term n(t) to the symbols output by the transmitter in Equation (7).

(7) r(t)=z(t)+n(t)

In the Rayleigh fading channel, a series of complex fading coefficients $a\left(t\right)=\frac{1}{\sqrt{2}}\left|a\right|$ are sampled from the complex standard normal distribution a∼CN(0,1). These coefficients are applied to scale the transmitter symbols before adding the noise term in Equation (8).

(8) r(t)=a(t)z(t)+n(t)

The Rician fading channel has the same structure as Equation (8), except that the fading coefficients are drawn from a parameterised complex normal distribution. The mean $\mu =\sqrt{K/(2(K+1))}$ and standard deviation $\sigma =\sqrt{1/(2(K+1))}$ are both determined by the Rician factor *K*, which in our simulations we define as K=10. The coefficients are then drawn from the complex normal distribution $a∼CN(\mu ,\sigma^{2})$ and applied to scale the transmitter symbols.

The PA-AWGN assumes a Rapp model of a solid state high power amplifier (SSPA) [37] that is applied to the output of the transmitter in Equation (9). The parameters for the model include the limiting output amplitude $A_{0}$, a gain parameter ν, and a smoothness parameter *p*; in our simulations, we configure $A_{0}=1$, ν=1, and p=5. The nonlinearity operates on the magnitude of the transmitter output, and is multiplied by the complex exponent of the argument of the transmitter output A=|z(t)|. In the PA-AWGN channel, AWGN is applied after amplification.

(9) $$\begin{array}{c} g\left(A\right)=\nu \frac{A}{{\left(1+{\left[{\left(\frac{\nu A}{A_{0}}\right)}^{2}\right]}^{p}\right)}^{1/2p}}\\ z^{\prime }\left(t\right)=g\left(|z\left(t\right)|\right)e^{j∠z(t)} \end{array}$$

The noise term n(t) in each of the channel models above is drawn from the complex normal distribution. When simulating the channel function, we define the desired level of SNR or ratio of energy per bit to the noise $E_{b}/N_{0}$ provided in dB. As the models learn a coded modulation with a code rate K/N, we convert this quantity to the ratio of energy per symbol to noise $E_{s}/N_{0}\;\mathrm{dB}=E_{b}/N_{0}\;\mathrm{dB}+10log_{10}\left(K/N\right)$ and use the linear ratio $E_{s}/N_{0}=10^{E_{s}/N_{0}\;\mathrm{dB}/10}$ to separate terms for $E_{s}$ and $N_{0}$. The term $E_{s}$ is estimated directly from the *L* transmitter IQ symbols $E_{s}=\sum_{t=1}^{L}z{\left(t\right)}^{2}/L$ and $N_{0}=E_{s}/\left(E_{s}/N_{0}\right)$. The noise is then sampled from the complex normal distribution $n\left(t\right)∼CN\left(0,\sigma^{2}\right)$ using the variance $\sigma^{2}=N_{0}/2$.

## 4. Results and Discussion

In this section, we evaluate the proposed method in the AWGN, Rician and Rayleigh fading, and PA-AWGN channels. In the AWGN channel, we train and compare the joint model and the proposed disjoint model for the 8 bit uncoded and Hamming(7,4) code rates. We additionally draw comparisons between receiver tuning for the joint model and the disjoint model. Receiver tuning is performed by training the joint model in the Rician fading channel and tuning the receiver in the Rayleigh fading channel. We make comparisons with receiver tuning by training the joint model on the AWGN channel and tuning the receiver in the PA-AWGN channel. This is performed for both code rates. We present a comparison between the proposed disjoint training method requiring feedback against the training without feedback. These results are reported in the Rayleigh fading channel. In addition, we present results for quantisation of the feedback, which can reduce the overall channel usage required during training.

**Figure 5:**
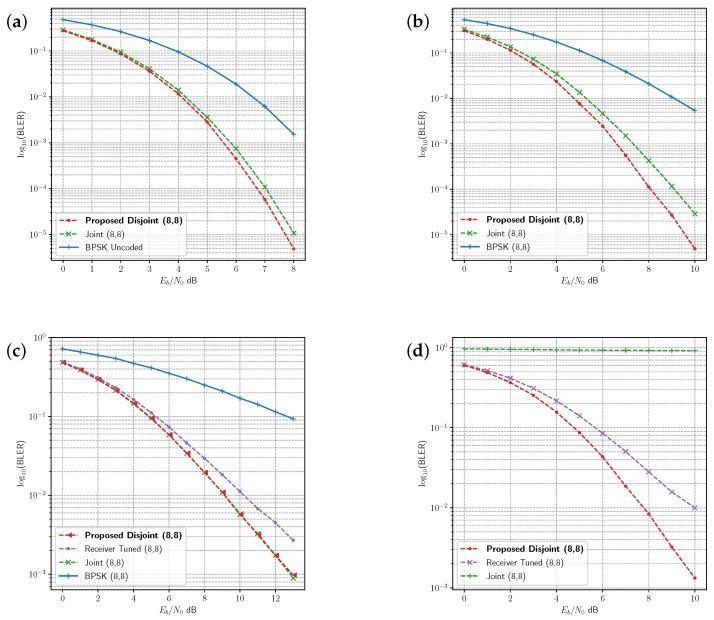
Comparison of BLER in the four channel environments for the uncoded 8 bit message. The joint learning, disjoint learning and BPSK modulation are compared in the (**a**) AWGN channel, (**b**) Rician fading channel, and (**c**) Rayleigh fading-channel. (**d**) Comparison between joint learning, disjoint learning, and receiver tuning in AWGN with PA-AWGN non-linearity.

The joint and disjoint learning methods for the 8 bit message are compared with uncoded binary phase shift keying (BPSK) under several channels in Figure 5. The proposed disjoint learning process provides slightly better performance than the joint learning procedure under AWGN (Figure 5a). In the Rician fading channel, disjoint learning achieves lower block error rate (BLER) than the joint learning method (Figure 5b), whereas disjoint and joint learning produce similar BLER in the Rayleigh fading channel (Figure 5c). Receiver tuning leverages the joint dense network from the Rician fading channel and updates the receiver under the Rayleigh fading channel (Figure 5c). Receiver tuning does not reach the same level of BLER as the other methods.

**Figure 6:**
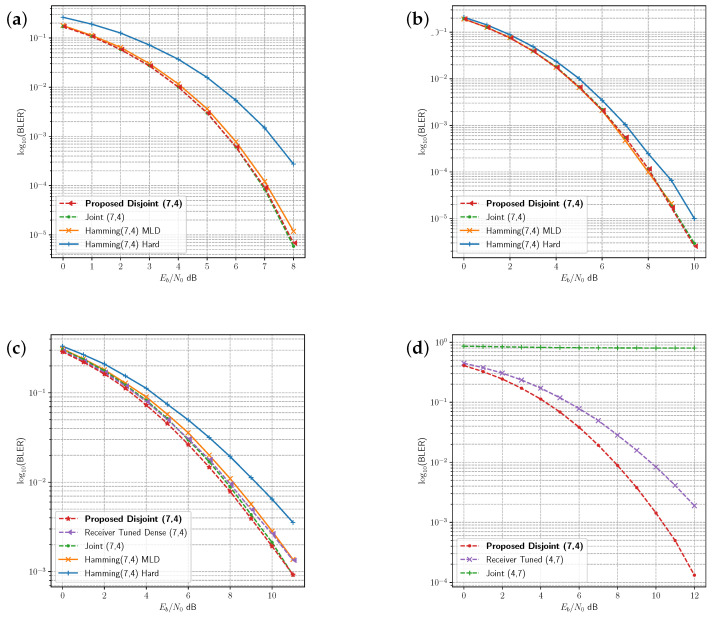
Comparison of BLER in the four channel environments for the K=4, N=7 code rate. The joint learning, disjoint learning and the BPSK modulated Hamming(7,4) code are compared in the (**a**) AWGN channel, (**b**) Rician fading channel, and (**c**) Rayleigh fading-channel. (**d**) Comparison between joint learning, disjoint learning, and receiver tuning in AWGN with PA-AWGN non-linearity.

Joint and disjoint learning methods are compared to the Hamming(7,4) code in Figure 6 in the AWGN (Figure 6a), Rician (Figure 6b), and Rayleigh (Figure 6c) fading channels. Both the joint and disjoint methods exhibit very similar or slightly better performance as maximum likelihood decoding (MLD) for the Hamming(7,4) code in each of these channels. Receiver tuning is repeated for the (7,4) code in Figure 6c, adapting the joint model receiver trained under Rician fading to the Rayleigh fading channel. While the performance is close to the other codes, it does not achieve the same BLER as the disjoint method with the transmitter optimised for the channel environment.

There is a difference in architecture between the joint and proposed disjoint models for the transmitter and receiver described in Section 3.2. The combination of the residual connections and additional dense layers increases the size of the disjoint models slightly, and contribute to the gain over the joint model. In comparison to uncoded BPSK modulation, the joint and proposed models learn a continuous code that is non-zero in both IQ coordinates; the resulting code is more complex than BPSK modulation, which is non-zero on the in-phase (I) axis. The performance of a code is related to the minimum squared distance between all codes [38]. Ideally, the transmitter should learn a code that has a large minimum Euclidean distance. Taking for example the K=4,N=7 learned code, we can compute the minimum ($d_{E_{min}}$), mean ($E[d_{E}]$), and variance ($Var[d_{E}]$) of the Euclidean distances for each of the proposed K=4,N=7 disjoint models, as shown in Table 3. The reference Hamming(7,4) code with a minimum binary distance ($d_{min}$) of 3 is included for comparison. The disjoint model has learned a slightly different code under each of the channels, each with a slightly different value for $d_{E_{min}}$. While $d_{E_{min}}$ is not always larger than the computed value for the Hamming(7,4) code, $E[d_{E}]$ is slightly larger, and the $Var[d_{E}]$ is quite low in comparison. We would expect that the learned code would perform slightly better in those channels where $d_{E_{min}}$ is larger than the reference code, which is indeed the case for AWGN. While the learned code in the Rayleigh channel has a slightly lower minimum Euclidean distance, it appears that the $E[d_{E}]$ and low $Var[d_{E}]$ may contribute to the overall performance of the learned code.

To further investigate the effect of the channel on tuning and disjoint learning, we compared the joint model trained on AWGN with a receiver tuned model and the disjoint model under the PA-AWGN channel. Figure 5d and Figure 6d show the BLER for the uncoded 8 bit message and the 4/7 code rate, respectively. In both cases, the AWGN joint model is unable to provide decoding for learned symbols under the PA-AWGN channel. However, the receiver tuned model derived from the same joint AWGN model learns to optimise the receiver, allowing it to classify messages in this environment. The advantage of the proposed disjoint learning algorithm is indicated by the improvement in performance over the receiver tuned model due to training both the transmitter and receiver.

**Figure 7:**
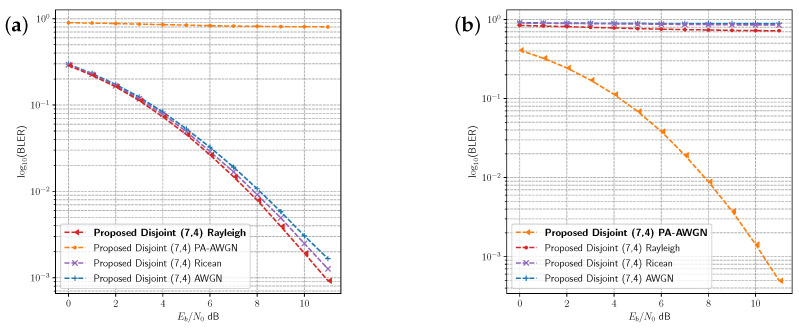
Comparison of BLER performance for the proposed disjoint models without retraining on targeted channel environments. Indicating the transmitter learns a code that is optimised for the targeted channel. (**a**) Comparison of BLER performance without retraining in the Rayleigh fading channel. (**b**) Comparison of BLER performance without retraining in the PA-AWGN channel.

Because the proposed disjoint model outperforms the receiver tuned model, it is clear that the transmitter model is learning a code that is specifically optimised to the target channel environment where it is trained. This is evident in the distance measurements of the K=4,N=7 code presented in Table 3. To evaluate the difference between the learned codes, we computed the BLER performance for models which were not trained on two of the selected channel environments. Figure 7a presents the BLER for the disjoint models which were not trained on the Rayleigh fading channel in comparison with the optimal disjoint model for that channel. The performance of the disjoint models optimised for the AWGN and Rician fading channel are similar to, but do not exactly match, the same performance of the optimised Rayleigh fading model. These two models have been optimised for slightly simpler channels than the Rayleigh fading channel. The Rician fading channel has slightly different fading characteristics from the Rayleigh fading channel, and the Rician model is closer in performance. The AWGN channel has no fading effects, and the resulting model has higher BLER than both of the other fading models. However, there is a large difference between the performance of the disjoint model optimised for the PA-AWGN channel and the other models. The performance is reversed in Figure 7b, where the PA-AWGN model is the optimal model. By imitating the remote receiver, the local channel/receiver enables the transmitter to learn codes which are optimised for the channel environment and which can be applied in channels with similar characteristics. However, it is possible for channel environments to differ significantly, as illustrated in Figure 7. The nonlinear effects of the amplifier are unique to the PA-AWGN channel, and are not shared with the other channels. In a practical wireless communications system, it is necessary to detect when the channel changes significantly (i.e., when performance degrades) and to either adapt using OAL and/or develop DL methods for adaptive modulation and coding schemes [38] that can select from multiple learned codes.

**Figure 8:**
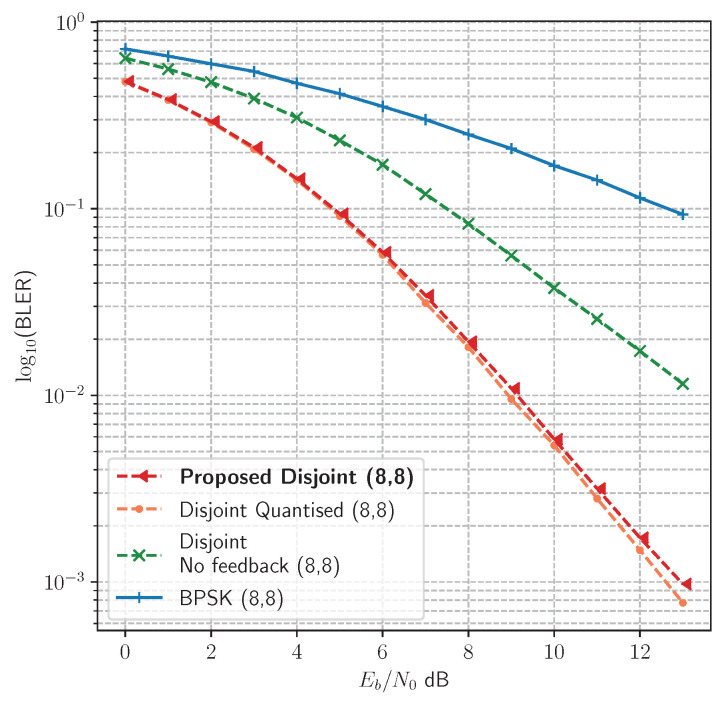
Comparison between learning without feedback, learning with soft values, and learning with quantised values in the Rayleigh fading channel.

The proposed method enables the transmitter to learn codes that are optimised during training for the observed channel environment. However, the question arises as to what extent imitating the remote receiver is helpful in achieving optimisation at the transmitter. Is it possible to achieve the same optimisation by simply training the local receiver against the true target message? We compared this no-feedback approach against the disjoint Learning method in Figure 8, where disjoint learning with feedback strongly outperforms learning without feedback. It is not sufficient to train the local receiver against a noiseless channel; instead, by imitating the remote receiver, enough information about the channel distortion is provided to the transmitter model during backpropagation to enable it to learn optimal symbols for the current channel condition. This is clearly indicated in both Figure 5 and Figure 6, where the disjoint method either outperforms or matches the joint learning method, achieving optimal BLER (in the case of the Hamming(7,4) code).

Feedback of soft values during disjoint training does require a large amount of data, depending on the message size; for example, in an uncoded 8 bit message, the feedback stage requires a batch size of 320×256 soft values. It is desirable to reduce the amount of information that needs to be sent over the feedback channel during learning. One possible method is to simply take the argmaxp(y|z) output at the remote receiver and feed back the integer indices for learning at the local channel/receiver; this reduces the amount of data to the batch size (320×1). As these integer values can be translated to a one-hot encoding on the transmitter side, the local channel/receiver then learns to imitate the remote receiver through the cross-entropy loss. Figure 8 compares the performance resulting from training with reduced information (Disjoint Quantised) as opposed to soft values (Proposed Disjoint), and indicates no loss of performance under the Rayleigh fading channel.

Our results show that the learning process in the transmitter is dependent on the local channel/receiver model. This is indicated by the ability to learn an equivalent or better performing code than the joint AE as well as by the difference in performance in different channels. The feedback of the estimates p(y|r) from the remote receiver contains implicit information about the channel environment. This implicit information is conveyed by the errors made at the remote receiver, which can be regarded as a kind of classification label noise, such as the type of regularisation introduced in [28]. Hence, by learning to imitate the remote receiver, the local channel/receiver learns to make the same errors over the course of learning. Unlike traditional supervised learning for classification, in which a model is optimised against a static set of target labels, the proposed learning process gradually changes all three models (the local transmitter, local channel/receiver, and remote receiver). The implication is that all three models are jointly optimised. In order to improve performance at the remote receiver, the transmitter alters the learned code based on the distance between the local channel/receiver estimate p(y|z) and the remote receiver estimate p(y|r). The need for backpropagation over an unknown channel is mitigated, as the information required to learn an optimal code is contained in the feedback of the estimates for p(y|r) from the remote receiver.

While we have demonstrated equivalent or better performance compared to the joint model, our work has a number of limitations. First, we assumed perfect synchronisation and did not apply matched filtering or any timing, phase, or frequency distortions. Second, for the purposes of discussion, we have limited our study to the domain of short codes. Third, the method requires high use of a feedback channel, similar to the RL-based methods. However, we have shown that it is possible to reduce the feedback channel usage; instead of learning to approximate the soft values for p(y|r) estimated at the remote receiver, it is possible to train against the argmaxp(y|r) without loss of performance. Finally, our method does not explicitly model the channel in the way that a GAN provides a separate channel model which can be reused outside of the training process. Instead, the local channel/receiver provides an implicit distortion to the transmitter in order to enable optimisation. Our approach represents a simplification over other training methods, requiring fewer models than the GAN approach by omitting the generator and discriminator models. The proposed method is able to take advantage of backpropagation directly, as opposed to the gradient approximation applied in RL methods, and does not require a complex coordinating protocol such as the one used in cooperative multi-agent learning.

## 5. Conclusions and Further Research Work

To date, disjoint learning methods have focused on simulation of the channel via GAN or gradient approximation through finite difference methods or RL approaches. In this paper, we have presented an additional approach to disjoint learning by learning to imitate the remote receiver. We have demonstrated equivalent performance to joint learning in AWGN, Rician, and Rayleigh fading channels, and shown that learning to imitate has the advantage of optimising both the transmitter and receiver, as opposed to receiver tuning, which can only adapt the remote receiver after joint learning. By comparing the distance metrics for learned codes, the performance of models in different channels, and the difference in performance between training with and without feedback, we have provided evidence that our proposed method is able to optimise the local transmitter and remote receiver models without an assumed channel model. The local channel/receiver model has no explicit knowledge of the channel, and by imitating the remote receiver, provides enough implicit channel information to enable the transmitter to learn optimal codes for the channel environment.

The limitations described in Section 4 provide an opportunity for future investigation. The assumption of perfect synchronisation can be addressed by incorporating additional channel perturbations and matched filtering. Further investigation into longer codes can be facilitated by incorporating bitwise estimation and concatenated codes. Bitwise estimation differs from symbolwise classification, and may alter the optimisation of the local channel/receiver during training. This method can find potential applications in joint-source coding and semantic coding, which have both benefited from the use of AE. In order to address changing channel conditions, it is possible to investigate alterations to the architecture to support adaptive modulation and coding schemes, or alternately to determine how to appropriately retrain the transmitter and receiver models under changing channel environments. In addition, future scope remains for investigating real-time training requirements over physical hardware. Even though this method makes no assumptions about the channel model, it is possible to train the models offline and adapt both transmitter and receiver models in a deployed scenario as opposed to receiver only tuning.

As an alternative to channel approximation and gradient approximation methods in disjoint learning, learning to imitate the remote receiver provides an additional method of disjoint learning without an assumed channel, and offers a simplified training procedure that can be applied to over-the-air learning in wireless communication systems.

## Figures and Tables

**Table 1:** The transmitter consists of four groups: input, skip block, a linear transformation, and an output block. The number of units are specified for the dense layers, while batch normalisation and swish activation preserve the same dimension of output as produced by the dense layer. A larger dimension of units was required for the 4/7 code rate as opposed to uncoded 8 bit message due to the coding gain required to match the Hamming(7,4) code.

Layer	Units Code Rate 7/4	Units Uncoded 8 Bit	Group
Input layer	$2^{K}$	$2^{K}$	Input
Dense layer	256	256	Skip block
Batch normalisation	-	-	
Swish activation	-	-	
Dense layer	128	16	
Batch normalisation	-	-	
Swish activation	-	-	
Dense layer	256	256	
Batch normalisation	-	-	
Swish activation	-	-	
Dense layer	2N	2N	2N linear block
Linear activation	-	-	
Reshape [N,2] layer	-	-	
Dense layer	2	2	Output [N,2]
Tanh activation	-	-	
Energy normalisation	-	-	

**Table 2:** The receiver network, consisting of three groups for input, feature learning (skip block), and output. The dimension of units are shown for each dense layer, with subsequent layers producing the same shape output as the preceding dense layer. A larger network was required to achieve the 4/7 code rate as opposed to the uncoded 8 bit message.

Layer	Units Code Rate 7/4	Units Uncoded 8 Bit	Group
Input layer	[N,2]	[N,2]	Input
Flatten layer	-	-	
Dense layer	256	256	Skip block
Batch normalisation	-	-	
Swish activation	-	-	
Dense layer	128	16	
Batch normalisation	-	-	
Swish activation	-	-	
Dense layer	256	256	
Batch normalisation	-	-	
Swish activation	-	-	
Dense layer	$2^{K}$	$2^{K}$	Output
Softmax activation	-	-	

**Table 3:** Computed minimum ($d_{E_{min}}$), mean ($E[d_{E}]$), and variance ($Var[d_{E}]$) of the Euclidean distances between $2^{K}$ messages. Distance measures are shown for the reference Hamming(4,7) and the disjoint K=4,N=7 code trained in each channel environment. The minimum binary distance ($d_{min}$) is provided for the Hamming code, but is not applicable for the learned continuous codes.

Code	$d_{\min}$	$d_{E_{\min}}$	$E[d_{E}]$	$\mathrm{Var}[d_{E}]$
Hamming(7,4)	3	3.46	3.83	0.21
Disjoint AWGN (7,4)	-	3.51	3.85	0.08
Disjoint Rician (7,4)	-	3.44	3.85	0.07
Disjoint Rayleigh (7,4)	-	3.37	3.86	0.05
Disjoint PA-AWGN (7,4)	-	3.06	3.85	0.08

## Data Availability

Data are contained within the article.
